# Chemical Constituents from *Flueggea virosa* and the Structural Revision of Dehydrochebulic Acid Trimethyl Ester

**DOI:** 10.3390/molecules21091239

**Published:** 2016-09-16

**Authors:** Chih-Hua Chao, Ying-Ju Lin, Ju-Chien Cheng, Hui-Chi Huang, Yung-Ju Yeh, Tian-Shung Wu, Syh-Yuan Hwang, Yang-Chang Wu

**Affiliations:** 1 School of Pharmacy, China Medical University, Taichung 40402, Taiwan; 2 Chinese Medicine Research and Development Center, China Medical University Hospital, Taichung 40447, Taiwan; 3 School of Chinese Medicine, China Medical University, Taichung 40402, Taiwan; yjlin@mail.cmu.edu.tw; 4 Genetic Center, Department of Medical Research, China Medical University Hospital, Taichung 40447, Taiwan; 5 Department of Medical Laboratory Science and Biotechnology, China Medical University, Taichung 40402, Taiwan; jccheng@mail.cmu.edu.tw (J.-C.C.); star740101@gmail.com (Y.-J.Y.); 6 Department of Chinese Pharmaceutical Sciences and Chinese Medicine Resources, China Medical University, Taichung 40402, Taiwan; hchuang@mail.cmu.edu.tw; 7 Department of Pharmacy, National Cheng Kung University, Tainan 70101, Taiwan; tswu@mail.ncku.edu.tw; 8 Department of Pharmacy and Graduate Institute of Pharmaceutical Technology, Tajen University, Pingtung 90741, Taiwan; 9 Endemic Species Research Institute, Council of Agriculture, Nantou 55244, Taiwan; son757780@yahoo.com.tw; 10 Center for Molecular Medicine, China Medical University Hospital, Taichung 40447, Taiwan; 11 Graduate Institute of Natural Products, Kaohsiung Medical University, Kaohsiung 80708, Taiwan

**Keywords:** *Flueggea virosa*, dehydrochebulic acid trimethyl ester, *ent*-podocarpane, anti-HCV, HCVcc

## Abstract

In an attempt to study the chemical constituents from the twigs and leaves of *Flueggea virosa*, a new terpenoid, 9(10→20)-*abeo*-*ent*-podocarpane, 3β,10α-dihydroxy-12-methoxy-13- methyl-9(10→20)-*abeo*-*ent*-podocarpa-6,8,11,13-tetraene (**1**), as well as five known compounds were characterized. Their structures were elucidated on the basis of spectroscopic analysis. In addition, the structure of dehydrochebulic acid trimethyl ester was revised as (2*S*,3*R*)-4*E*-dehydrochebulic acid trimethyl ester based on a single-crystal X-ray diffraction study. The in vitro anti-hepatitis C virus (anti-HCV) activity and cytotoxicity against Huh7.5 cells for the isolated compounds were evaluated.

## 1. Introduction

**Chart 1:**
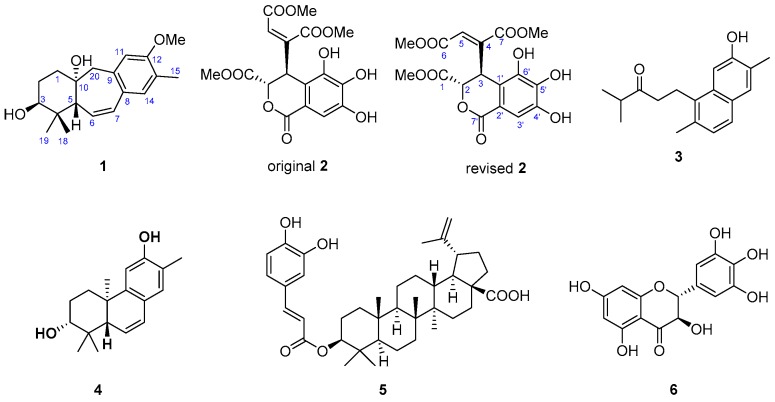
Structures of compounds **1**–**6**.

*Flueggea virosa* (Roxb. ex Wild) Baill, belonging to the Euphorbiaceae family, is a common medicinal plant in Africa and China [1,2]. In previous reports, *Securinega* alkaloids have been widely regarded as a representative group of the genus *Flueggea* [3], formerly classified as *Securinega* genus. However, our prior studies have demonstrated that the roots of *F. virosa* contained a series of non-alkaloids, including *ent*-podocarpanes [4,5], 9(10→20)-*abeo*-*ent*-podocarpanes [6], 3,4-*seco*-*ent*-podocarpanes [7], and 3,4-*seco*-30-*nor*-friedelanes [6], some of which were endowed with anti-HCV activities and weak toxicities. Among them, 4-hydroxy-12-methoxy-13-methyl-3,4-*seco*-*ent*-podocarpa-6,8,11,13-tetraen-3-oic acid was found to be a brand new type anti-HCV agent [7]. The non-alkaloids in the other parts of this plant still received less attention. Thus, the isolation of active constituents from the twigs and leaves of *F. virosa* was carried out, which resulted in the isolation of a new 9(10→20)-*abeo*-*ent*-podocarpane, namely 3β,10α-dihydroxy-12-methoxy-13-methyl-9(10→20)-*abeo*-*ent*-podocarpa-6,8,11,13-tetraene (**1**), alone with five known compounds. In addition, the structural revision of dehydrochebulic acid trimethyl ester (Chart 1, original **2**) was also reported in this study.

## 2. Results and Discussion

The MeOH extract from the twigs and leaves of *F. virosa was* concentrated and the alkaloids were removed by partition with acidic water. The resulting nonalkaloid layer was separated repeatedly by column chromatography to afford a new 9(10→20)-*abeo*-*ent*-podocarpane (**1**) and five known compounds, which were identified as 4*E*-dehydrochebulic acid trimethyl ester (**2**) [8,9], 12-hydroxy-20(10→5)-*abeo*-4,5-*seco*-podocarpa-5(10),6,8,11,13-pentaen-3-one (**3**) [10], 3β,12-dihydroxy-13-methylpodocarpa-6,8,11,13-tetraene (**4**) [10], betulinic acid 3β-calfeate (**5**) [11], and (+) ampelosin E (**6**) [12].

**Figure 1:**
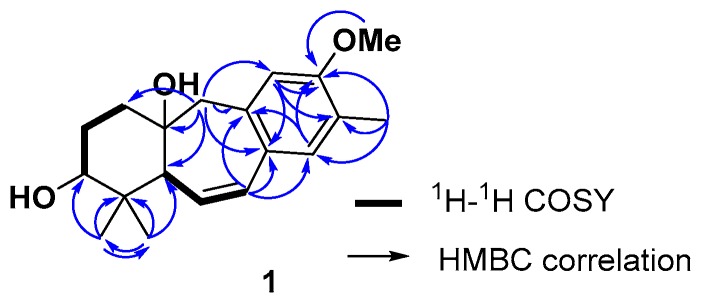
Selected ^1^H−^1^H COSY (▬) and HMBC (→) correlations of **1**.

**Figure 2:**
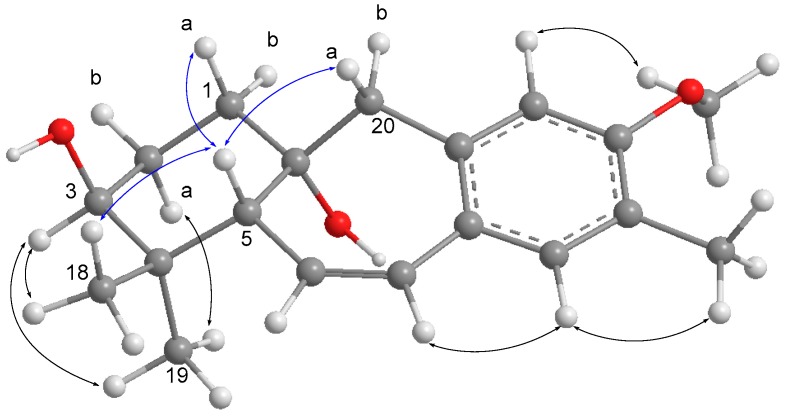
Selected NOE correlations of **1**.

Compound **1** was obtained as a colorless oil and a molecular formula of C_19_H_26_O_3_ was deduced for this compound based on the molecular ion peak [M − H]^−^ at *m*/*z* 301.1805 in the (−)-HR-APCI-MS (calcd for C_19_H_25_O_3_, 301.1809). Inspection of the overall ^1^H- and ^13^C-NMR data revealed that it is a member of 9(10→20)-*abeo*-*ent*-podocarpanes [6], with slight chemical shift differences for resonances of substituent patterns. The 12-methoxy group was readily assigned from its proton chemical shift at δ_H_ 3.82 (s), while the 6,7-double bond was deduced according to the proton chemical shifts at δ_H_ 6.58 (1H, dd, *J* = 12.1, 2.6 Hz) and 5.78 (1H, dd, *J* = 12.1, 4.4 Hz) (Table 1) [6]. The above assignment was confirmed by the interpretation of ^1^H–^1^H COSY and HMBC correlations (Figure 1). The 3β-OH was determined based on the chemical shift and coupling patterns of H-3 [δ_H_ 3.51 (1H, t, *J* = 2.8 Hz)]. The relative configuration of **1** was further confirmed by the analysis of NOE correlations (Figure 2). The NOE correlations of H-5/H-20a, H-5/H-1a, and H-5/H_3_-18 confirmed that **1** possessed the same relative configurations (10α-OH, 5β-H) as those of 9(10→20)-*abeo*-*ent*-podocarpanes isolated previously from the roots of *F. virosa* [6] Accordingly, the structure of **1** was determined as 3β,10α-dihydroxy-12-methoxy-13-methyl-9(10→20)-*abeo*-*ent*-podocarpa-6,8,11,13-tetraene.

**Figure 3:**
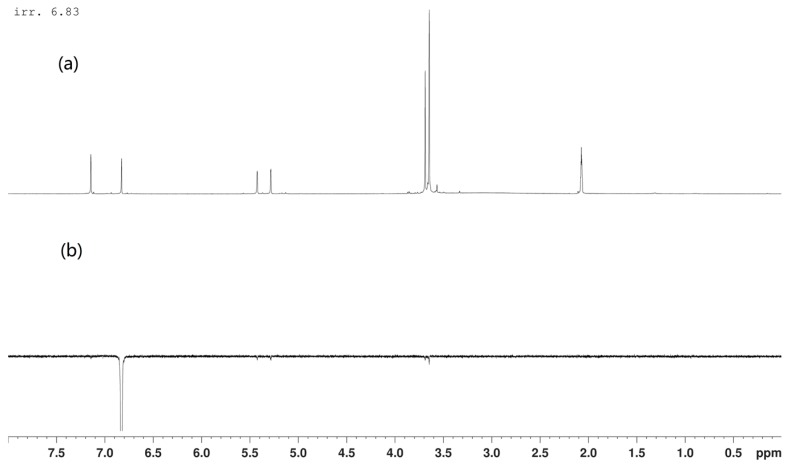
(**a**) ^1^H-NMR spectrum of **2** measured in acetone-*d*_6_ and (**b**) selective 1D NOESY spectrum of **2**: selective irradiation of H-5 (δ_H_ = 6.83 ppm) using *selnogp.3* Bruker program (parameter set: ns 128, p12 = 80 ms, and d8 = 400 ms).

**Figure 4:**
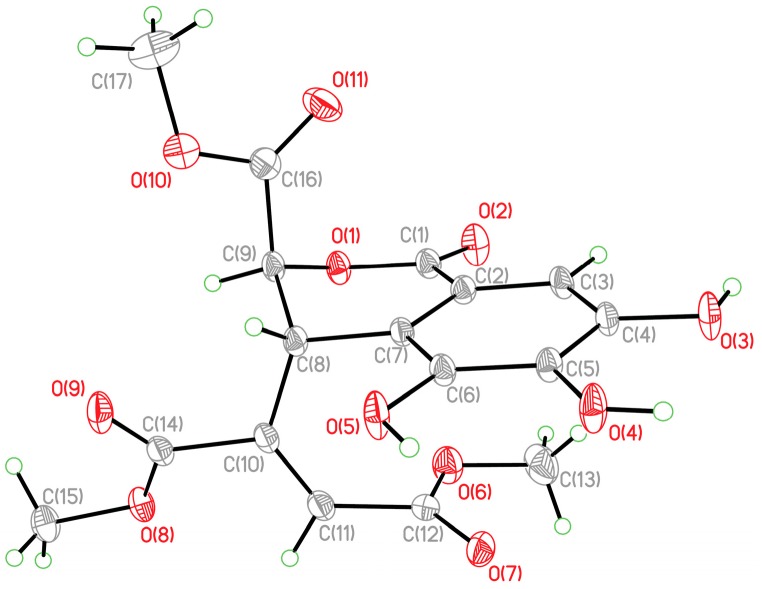
X-ray ORTEP drawing of **2**.

The structure of dehydrochebulic acid trimethyl ester (original **2**, Chart 1) with a *cis*-methylbutenedioate scaffold was reported from *Phyllanthus urinaria* by Yao et al. in 1993 [9]. However, the authors proposed the wrong geometry for the methylbutenedioate moiety because of failure to compare the NMR data for this moiety with those reported in the literature. The ^1^H- and ^13^C-NMR data of **2** measured in acetone-*d*_6_ and pyridine-*d*_6_ were in good agreement with those reported in the literature [8,9]. In the selective 1D NOESY experiment (Figure 3), irradiation of olefinic H-5 did not show enhancements with other signals, suggesting that the 4,5-double bond in **2** has a *trans* geometry. A single-crystal X-ray analysis was performed on **2** (Figure 4), which led the absolute configurations to be defined as 2*S*,3*R* according to the value of Flack parameter 0.09 (14), and confirmed the geometry of a 4*E*-double bond. Accordingly, the structure of **2** was revised as (2*S*,3*R*)-4*E*-dehydrochebulic acid trimethyl ester (Chart 1, revised **2**).

In order to continue our exploration for anti-HCV agents from a natural source, the isolated compounds were subjected to anti-HCV and cytotoxic evaluation. As shown in Table 2, the anti-HCV activity of compounds **1**–**6** was assayed using the cell-based HCV cell culture (HCVcc) infection system, while the toxicity toward human hepatoma Huh7.5 cells was also measured using a 3-(4,5-dimethylthiazol-2-yl)-5-(3-carboxymethoxyphenyl)-2-(4-sulfophenyl)-2*H*-tetrazolium salt (MTS) assay. In addition, the therapeutic indices (TI = IC_50_/EC_50_) were also calculated to estimate their potency as anti-HCV agents. The result showed that compounds **3**–**5** possessed better potencies than honokiol (TI = 2.1) [13], a natural anti-HCV agent from *Magnolia officinalis* [14], with TI values of 6.8, 9.2, and 2.9, respectively.

## 3. Experimental Section

### 3.1. General Experimental Procedures

Melting point and optical rotation data were recorded on a Yanako MP-500P micro melting point apparatus (Yanaco, Kyoto, Japan) and a JASCO P2000 digital polarimeter (JASCO Corporation, Tokyo, Japan), respectively. IR spectra were recorded as a thin film on a KBr plate or using a conventional KBr pellet on a Shimadzu IR Prestige21 FT-IR spectrometer (Shimadzu, Milan, Italy). UV data were obtained using a Shimadzu UV-1700 UV/Vis spectrometer (Shimadzu). NMR spectra (400 MHz) were recorded using a Bruker Avance 400 spectrometer equipped with a 5-mm Dual *z*-gradient probe ^1^H/^13^C (Bruker, Rheinstetten, Germany). HR-APCI-MS and HR-ESI-MS were measured with an LTQ Orbitrap XL mass spectrometer (Thermo Fisher Scientific Corp., Waltham, MA, USA). Column chromatography (CC) was performed on silica gel 60 (230–400 mesh, Merck, Darmstadt, Germany) and RP-18 gel (230–400 mesh, SiliaBond C18, SiliCycle, Quebec City, QC, Canada). Silica gel plates (Kieselgel 60 F254, 0.25 mm, Merck) were used for thin-layer chromatography (TLC) analysis, and spots were visualized by spraying with 10% H_2_SO_4_ solution followed by heating.

### 3.2. Plant Material

Twigs and leaves of *F. virosa* were collected in North Pingtung, Taiwan, in August 2013. Botanical identification was performed by Dr. S.-Y. Hwang. A voucher specimen (chao-002) was deposited in the School of Pharmacy, China Medical University.

### 3.3. Extraction and Isolation

The twigs and leaves of *F. virosa* (35 kg) were crushed and extracted exhaustively with MeOH (5 × 40 L). The organic extract was concentrated to an aqueous suspension and was further partitioned between CHCl_3_ and H_2_O. The CHCl_3_ extract was washed with 3% aqueous tartaric acid three times to remove alkaloids. The resulting nonalkaloid extract (420 g) was fractionated by open column chromatography on silica gel using hexane/EtOAc and EtOAc/MeOH mixtures of increasing polarity to yield 14 fractions. Fraction 9, eluted with EtOAc/MeOH (9:1), was repeatedly separated by RP-18 column chromatography with gradient elution (MeOH/H_2_O, 5% to 40%) to yield **2** (200.2 mg) and **6** (12.1 mg). Fraction 7 was fractionated by silica gel column chromatography using gradient elution (hexane/EtOAc, 100:0 to 53:47) to afford 20 subfractions (7A to 7T). Compounds **1** (100.2 mg), **4** (266.1 mg), **3** (19.1 mg), and **5** (80.0 mg) were obtained from fraction 7L by repeated column chromatography over silica gel with (hexane/EtOAc, 100:0 to 60:40).

*3β,10α-Dihydroxy-12-methoxy-13-methyl-9(10→20)-abeo-ent-podocarpa-6,8,11,13-tetraene* (**1**): pale yellow oil; [*α*]${}_{D}^{25}$ +139 (*c* 1.65, CHCl_3_); UV (MeOH) λ_max_ (log ε) 214 (4.23), 261 (3.94) nm; IR (neat) *v*_max_ 3444, 3003, 2953, 2931, 2870, 1610, 1568, 1504, 1446, 1435, 1384, 1336, 1315, 1257, 1217, 1114, 1097, 1045, 1020, and 981 cm^−1^; for ^1^H- and ^13^C-NMR data in CDCl_3_, see Table 2; (−)-APCI-MS *m/z* 301 [M − H]^−^; (−)-HR-APCI-MS *m/z* 301.1805 [M − H]^−^ (calcd for C_19_H_25_O_3_, 301.1809).

*(2S,3R)-4E-Dehydrochebulic acid trimethyl ester* (**2**): colorless crystal; mp 216–218 °C (MeOH), [*α*]${}_{D}^{25}$ +15 (*c* 28.45, MeOH); UV (MeOH) λ_max_ (log ε) 226 (4.47), 287 (3.97) nm; IR (KBr) *v*_max_ 3394, 3257, 1761, 1752, 1730, 1708, 1649 1622, 1608, 1537, 1494, 1431, 1390, 1369, 1352, 1309, 1278, 1244, 1209, 1180, 1114, 1066, and 1006 cm^−1^.

### 3.4. Crystallographic Data of ***2***

A colorless crystal of **2** with sizes of 0.62 × 0.50 × 0.47 mm^3^ was obtained at room temperature by slow evaporation in MeOH solution. Diffraction intensity data were acquired with a CCD area detector with graphite-monochromated CuKα radiation (*λ* = 1.54178 Å). Crystal data for **2**: C_17_H_16_O_11_, *M* = 396.30, orthorhombic, *a* = 7.1567(2) Å, *b* = 9.7147(3) Å, *c* = 25.1934(7) Å, *α* = 90.00°, *β* = 90.00°, *γ* = 90.00°, *V* = 1751.58(9) Å^3^, *T* = 150(2) K, space group *P*2_1_2_1_2_1_, *Z* = 4, *μ*(CuKα) = 1.115 mm^−1^, 6642 reflections collected, 3372 independent reflections (*R_int_* = 0.0199). The final *R_1_* values were 0.0298 (*I* > 2*σ*(*I*)). The final *wR*(*F*^2^) values were 0.0787 (*I* > 2*σ*(*I*)). The final *R_1_* values were 0.0315 (all data). The final *wR*(*F*^2^) values were 0.0800 (all data). The goodness of fit on *F*^2^ was 1.028. Flack parameter = 0.09(14). Crystallographic data for this compound have been deposited with the Cambridge Crystallographic Data Centre (deposition number CCDC 1484756) (Supplementary Materials). Copies of the data can be obtained, free of charge, on application to the Director, CCDC, 12 Union Road, Cambridge CB21EZ, UK [fax: +44(0)-1223-336033 or e-mail: deposit@ccdc.cam.ac.uk].

### 3.5. Infection Inhibition Assay

The JC1-Luc2A HCV reporter viral particles were prepared as described previously [15,16]. In brief, in vitro-transcribed genomic JC1-Luc2A RNA was delivered into Huh7.5.1 cells by electroporation. The virus-containing supernatant was collected for 4 days, clarified by low-speed centrifugation, passed through a filter with a pore size of 0.45 μm, and quantitated by fluorescent focus assay. The MTS assay was used to determine the cell viability, while HCV infection inhibition assay was performed according to the published protocol in [6,15]. Huh7.5.1 cells were seeded into the 96-well culture plates at a cell density of 1 × 10^4^ cells/well for 24 h followed by incubation with HCV reporter virus (with 0.1 MOI) for an additional 4 h. After removing the virus containing supernatant, the cells were washed 3 times with PBS and then replaced with medium containing indicated compound for 72 h. The luciferase activity assay was performed by the collected cell lysates. Honokiol, a known natural product with anti-HCV activity, was used as an inhibition positive control.

## 4. Conclusions

A new 9(10→20)-*abeo*-*ent*-podocarpane (**1**) and five known compounds were isolated from the twigs and leaves of *F. virosa*. The structures of the isolates were identified on the basis of NMR, MS, and IR spectroscopic data. The structure 4*E*-dehydrochebulic acid trimethyl ester was revised and its absolute configuration was determined for the first time. Comparing the present study with our prior investigation [4,5,6,7], it was found that the diversity in chemical constitutions of the roots were quite different from those of the twigs and leaves of *F. virosa*. Furthermore, 3β,12-dihydroxy-13-methylpodocarpa-6,8,11,13-tetraene (**4**) was found to be abundant in the twigs and leaves; however, the 12-*O*-methyl analogue of **4** was isolated as the main constituent in the roots [4]. Although the 9(10→20)-*abeo*-*ent*-podocarpane skeleton had been reported from the roots of this plant [6], it was still rare in a natural source. The present anti-HCV evaluation suggested that the *ent*-podocarpane derivatives might be the active ingredients in this plant.

## Figures and Tables

**Table 1:** ^1^H- and ^13^C-NMR Spectroscopic Data (400 and 100 MHz, resp.; CDCl_3_) of **1**.

	1		
	δ_H_ (*J* in Hz)	δ_C_ (Mult.)	HMBC
1	2.05 td (14.1, 4.4)	33.0 (CH_2_)	2, 3, 20
	1.61 m		
2	2.27 m	25.5 (CH_2_)	1
	1.62 m		
3	3.51 t (2.8)	75.6 (CH)	1, 5
4		38.2 (C)	
5	2.45 dd (4.4, 2.6)	49.6 (CH)	4, 6, 7, 10, 18, 19
6	5.78 dd (12.1, 4.4)	128.0 (CH)	4, 5, 8, 10
7	6.58 dd (12.1, 2.6)	131.3 (CH)	5, 9, 14
8		128.4 (C)	
9		135.2 (C)	
10		76.4 (C)	
11	6.56 s	112.2 (CH)	8, 12, 13, 20
12		156.9 (C)	
13		124.4 (C)	
14	6.95 s	132.4 (CH)	7, 9, 12
15	2.17 s	15.6 (CH_3_)	12, 13, 14
18	1.00 s	27.2 (CH_3_)	3, 4, 5, 19
19	1.09 s	22.4 (CH_3_)	3, 4, 5, 18
20	2.96 d (14.2)	51.6 (CH_2_)	1, 5, 8, 9, 10, 11
	2.68 d (14.2)		
12-OMe	3.82 s	55.3 (CH_3_)	12

**Table 2:** Anti-HCV activities (EC_50_) and cytotoxicity (IC_50_) of **1**–**6**.

Compound	EC_50_ (μM) ^a^	IC_50_ (μM) ^b^	(TI) ^c^
**1**	27.4 ± 1.4	>100	- ^d^
**2**	98.4 ± 2.1	>100	- ^d^
**3**	12.8 ± 3.1	87.1 ± 5.1	6.8
**4**	7.7 ± 2.7	70.5 ± 4.2	9.2
**5**	20.8 ± 2.0	60.7 ± 2.0	2.9
**6**	66.7 ± 1.8	>100	- ^d^
honokiol	22.4 ± 0.5	48.8 ± 0.8	2.1

^a^ EC_50_: concentration that inhibits HCVcc infection by 50%. ^b^ IC_50_: concentration that inhibits cell growth by 50%. ^c^ Therapeutic index (TI) = IC_50_/EC_50_. ^d^ not calculated.

## Supplementary Materials

Supplementary materials can be accessed at: http://www.mdpi.com/1420-3049/21/9/1239/s1.
