# Supplementary material for: Chemical Constituents from Flueggea virosa and the Structural Revision of Dehydrochebulic Acid Trimethyl Ester
Source: Molecules. 2016 Sep 16;21(9):1239. doi: 10.3390/molecules21091239 (PMC6274521; doi:10.3390/molecules21091239)
Supplement: Supplementary file 1 [file molecules-21-01239-s001.pdf]

Supplementary Materials: Chemical Constituents from *Flueggea virosa* and the Structural Revision of Dehydrochebulic Acid Trimethyl Ester

Chih-Hua Chao, Ying-Ju Lin, Ju-Chien Cheng, Hui-Chi Huang, Yung-Ju Yeh, Tian-Shung Wu, Syh-Yuan Hwang and Yang-Chang Wu

Table S1. Crystal data and structure refinement for fvs9r1.

Identification code	fvs9r1	
Empirical formula	C17 H16 O11	
Formula weight	396.30	
Temperature	150(2) K	
Wavelength	1.54178 Å	
Crystal system	Orthorhombic	
Space group	P 21 21 21	
Unit cell dimensions	a = 7.1567(2) Å	α = 90°
	b = 9.7147(3) Å	β = 90°
	c = 25.1934(7) Å	γ = 90°
Volume	1751.58(9) $Å^3$	
Z	4	
Density (calculated)	1.503 Mg/m^3	
Absorption coefficient	1.115 mm^{-1}	
F(000)	824	
Crystal size	0.62 × 0.50 × 0.47 mm^3	
Theta range for data collection	3.51 to 72.25°.	
Index ranges	$-8 \leq h \leq 8$, $-7 \leq k \leq 11$, $-30 \leq l \leq 27$	
Reflections collected	6642	
Independent reflections	3372 [R(int) = 0.0199]	
Completeness to theta = 72.25°	98.7%	
Absorption correction	Semi-empirical from equivalents	
Max. and min. transmission	1.00000 and 0.83165	
Refinement method	Full-matrix least-squares on F^2	
Data/restraints/parameters	3372/0/266	
Goodness-of-fit on F2	1.028	
Final R indices [I>2sigma(I)]	R1 = 0.0298, wR2 = 0.0787	
R indices (all data)	R1 = 0.0315, wR2 = 0.0800	
Absolute structure parameter	0.09(14)	
Extinction coefficient	0.0045(3)	
Largest diff. peak and hole	0.227 and −0.168 e.$Å^{-3}$	

Table S2. Atomic coordinates (×10⁴) and equivalent isotropic displacement parameters (Å² x 10³) for fvs9r1. U(eq) is defined as one third of the trace of the orthogonalized U^{ij} tensor.

	x	y	z	U(eq)
O(1)	4716(2)	10511(1)	2042(1)	19(1)
O(2)	5475(2)	8722(1)	2531(1)	26(1)
O(3)	3367(2)	4544(1)	1490(1)	26(1)
O(4)	2345(2)	5588(1)	521(1)	24(1)
O(5)	2358(2)	8376(1)	353(1)	25(1)
O(6)	8085(2)	8738(1)	1413(1)	27(1)
O(7)	7303(2)	7666(1)	656(1)	28(1)
O(8)	6780(2)	12838(1)	597(1)	28(1)
O(9)	3650(2)	12774(1)	509(1)	25(1)
O(10)	423(2)	11957(1)	1643(1)	29(1)
O(11)	1048(2)	10428(1)	2290(1)	33(1)
C(1)	4725(2)	9135(2)	2130(1)	18(1)
C(2)	3949(2)	8238(2)	1718(1)	16(1)
C(3)	3924(2)	6831(2)	1820(1)	16(1)
C(4)	3399(2)	5932(2)	1424(1)	17(1)
C(5)	2875(2)	6431(2)	924(1)	16(1)
C(6)	2874(2)	7850(2)	828(1)	16(1)
C(7)	3427(2)	8764(2)	1222(1)	15(1)
C(8)	3539(2)	10298(2)	1122(1)	16(1)
C(9)	3441(2)	11060(2)	1658(1)	17(1)
C(10)	5278(2)	10779(2)	825(1)	17(1)
C(11)	6860(2)	10105(2)	744(1)	20(1)
C(12)	7380(2)	8683(2)	925(1)	19(1)
C(13)	8741(3)	7450(2)	1641(1)	35(1)
C(14)	5118(2)	12233(2)	621(1)	20(1)
C(15)	6830(3)	14231(2)	393(1)	36(1)
C(16)	1484(2)	11078(2)	1907(1)	21(1)
C(17)	−1398(3)	12244(2)	1869(1)	37(1)

Table S3. Bond lengths [Å] and angles [°] for fvs9r1.

O(1)-C(1)	1.3545(18)
O(1)-C(9)	1.4330(19)
O(2)-C(1)	1.2130(19)
O(3)-C(4)	1.3599(18)
O(3)-H(3A)	0.82(3)
O(4)-C(5)	1.3583(19)
O(4)-H(4A)	0.87(3)
O(5)-C(6)	1.3542(18)
O(5)-H(5A)	0.80(2)
O(6)-C(12)	1.330(2)
O(6)-C(13)	1.454(2)
O(7)-C(12)	1.200(2)
O(8)-C(14)	1.328(2)
O(8)-C(15)	1.447(2)
O(9)-C(14)	1.208(2)
O(10)-C(16)	1.321(2)
O(10)-C(17)	1.449(2)
O(11)-C(16)	1.194(2)
C(1)-C(2)	1.464(2)

C(2)-C(3)	1.391(2)
C(2)-C(7)	1.400(2)
C(3)-C(4)	1.378(2)
C(3)-H(3B)	0.95
C(4)-C(5)	1.399(2)
C(5)-C(6)	1.399(2)
C(6)-C(7)	1.390(2)
C(7)-C(8)	1.514(2)
C(8)-C(10)	1.526(2)
C(8)-C(9)	1.541(2)
C(8)-H(8A)	1
C(9)-C(16)	1.535(2)
C(9)-H(9A)	1
C(10)-C(11)	1.324(2)
C(10)-C(14)	1.507(2)
C(11)-C(12)	1.501(2)
C(11)-H(11A)	0.95
C(13)-H(13A)	0.98
C(13)-H(13B)	0.98
C(13)-H(13C)	0.98
C(15)-H(15A)	0.98
C(15)-H(15B)	0.98
C(15)-H(15C)	0.98
C(17)-H(17A)	0.98
C(17)-H(17B)	0.98
C(17)-H(17C)	0.98
C(1)-O(1)-C(9)	118.72(12)
C(4)-O(3)-H(3A)	108.5(19)
C(5)-O(4)-H(4A)	108(2)
C(6)-O(5)-H(5A)	110.0(16)
C(12)-O(6)-C(13)	116.93(14)
C(14)-O(8)-C(15)	116.90(15)
C(16)-O(10)-C(17)	116.32(14)
O(2)-C(1)-O(1)	117.67(14)
O(2)-C(1)-C(2)	124.17(15)
O(1)-C(1)-C(2)	118.01(13)
C(3)-C(2)-C(7)	121.25(14)
C(3)-C(2)-C(1)	117.37(14)
C(7)-C(2)-C(1)	121.08(14)
C(4)-C(3)-C(2)	119.56(13)
C(4)-C(3)-H(3B)	120.2
C(2)-C(3)-H(3B)	120.2
O(3)-C(4)-C(3)	122.92(14)
O(3)-C(4)-C(5)	116.76(14)
C(3)-C(4)-C(5)	120.31(14)
O(4)-C(5)-C(4)	122.59(14)
O(4)-C(5)-C(6)	117.64(14)
C(4)-C(5)-C(6)	119.77(14)
O(5)-C(6)-C(7)	117.95(13)
O(5)-C(6)-C(5)	121.66(14)
C(7)-C(6)-C(5)	120.38(14)
C(6)-C(7)-C(2)	118.71(14)
C(6)-C(7)-C(8)	121.66(13)
C(2)-C(7)-C(8)	119.60(13)

C(7)-C(8)-C(10)	115.24(14)
C(7)-C(8)-C(9)	108.89(12)
C(10)-C(8)-C(9)	108.65(12)
C(7)-C(8)-H(8A)	107.9
C(10)-C(8)-H(8A)	107.9
C(9)-C(8)-H(8A)	107.9
O(1)-C(9)-C(16)	108.02(12)
O(1)-C(9)-C(8)	112.60(12)
C(16)-C(9)-C(8)	113.89(13)
O(1)-C(9)-H(9A)	107.3
C(16)-C(9)-H(9A)	107.3
C(8)-C(9)-H(9A)	107.3
C(11)-C(10)-C(14)	118.44(14)
C(11)-C(10)-C(8)	128.47(15)
C(14)-C(10)-C(8)	113.06(14)
C(10)-C(11)-C(12)	128.32(15)
C(10)-C(11)-H(11A)	115.8
C(12)-C(11)-H(11A)	115.8
O(7)-C(12)-O(6)	124.94(15)
O(7)-C(12)-C(11)	125.06(14)
O(6)-C(12)-C(11)	109.78(13)
O(6)-C(13)-H(13A)	109.5
O(6)-C(13)-H(13B)	109.5
H(13A)-C(13)-H(13B)	109.5
O(6)-C(13)-H(13C)	109.5
H(13A)-C(13)-H(13C)	109.5
H(13B)-C(13)-H(13C)	109.5
O(9)-C(14)-O(8)	125.12(14)
O(9)-C(14)-C(10)	123.61(15)
O(8)-C(14)-C(10)	111.25(14)
O(8)-C(15)-H(15A)	109.5
O(8)-C(15)-H(15B)	109.5
H(15A)-C(15)-H(15B)	109.5
O(8)-C(15)-H(15C)	109.5
H(15A)-C(15)-H(15C)	109.5
H(15B)-C(15)-H(15C)	109.5
O(11)-C(16)-O(10)	126.71(17)
O(11)-C(16)-C(9)	124.19(17)
O(10)-C(16)-C(9)	109.05(13)
O(10)-C(17)-H(17A)	109.5
O(10)-C(17)-H(17B)	109.5
H(17A)-C(17)-H(17B)	109.5
O(10)-C(17)-H(17C)	109.5
H(17A)-C(17)-H(17C)	109.5
H(17B)-C(17)-H(17C)	109.5

Symmetry transformations used to generate equivalent atoms.

Table S4. Anisotropic displacement parameters (Å^2 × 10^3) for fvs9r1. The anisotropic displacement factor exponent takes the form: $-2\pi^2[h^2 \cdot a^{*2}U^{11} + ... + 2\ h \cdot k \cdot a^* \cdot b^* \cdot U^{12}]$.

	**U^{11}**	**U^{22}**	**U^{33}**	**U^{23}**	**U^{13}**	**U^{12}**
O(1)	30(1)	12(1)	16(1)	−2(1)	−3(1)	−2(1)
O(2)	45(1)	17(1)	16(1)	−1(1)	−11(1)	−2(1)
O(3)	51(1)	9(1)	16(1)	2(1)	−9(1)	−2(1)
O(4)	44(1)	11(1)	18(1)	−2(1)	−11(1)	−1(1)
O(5)	50(1)	11(1)	14(1)	0(1)	−10(1)	1(1)
O(6)	33(1)	23(1)	24(1)	3(1)	−4(1)	1(1)
O(7)	47(1)	16(1)	23(1)	−1(1)	6(1)	4(1)
O(8)	36(1)	14(1)	34(1)	7(1)	10(1)	−1(1)
O(9)	39(1)	13(1)	24(1)	2(1)	−4(1)	2(1)
O(10)	27(1)	28(1)	31(1)	3(1)	4(1)	2(1)
O(11)	43(1)	31(1)	26(1)	4(1)	12(1)	−5(1)
C(1)	25(1)	14(1)	15(1)	−1(1)	0(1)	−2(1)
C(2)	21(1)	13(1)	13(1)	−1(1)	0(1)	0(1)
C(3)	26(1)	14(1)	10(1)	3(1)	−3(1)	0(1)
C(4)	23(1)	10(1)	17(1)	2(1)	−1(1)	−1(1)
C(5)	23(1)	11(1)	14(1)	−2(1)	−3(1)	−2(1)
C(6)	24(1)	13(1)	11(1)	2(1)	−1(1)	1(1)
C(7)	21(1)	11(1)	13(1)	0(1)	1(1)	−1(1)
C(8)	24(1)	10(1)	13(1)	−2(1)	0(1)	−1(1)
C(9)	27(1)	10(1)	14(1)	−1(1)	1(1)	0(1)
C(10)	30(1)	11(1)	11(1)	0(1)	1(1)	−2(1)
C(11)	30(1)	14(1)	16(1)	0(1)	3(1)	−3(1)
C(12)	22(1)	17(1)	18(1)	1(1)	5(1)	1(1)
C(13)	31(1)	36(1)	37(1)	16(1)	−3(1)	5(1)
C(14)	35(1)	12(1)	12(1)	−1(1)	4(1)	0(1)
C(15)	48(1)	13(1)	47(1)	9(1)	19(1)	−1(1)
C(16)	30(1)	14(1)	19(1)	−6(1)	4(1)	−4(1)
C(17)	26(1)	49(1)	37(1)	−9(1)	2(1)	4(1)

Table S5. Hydrogen coordinates (×10^4) and isotropic displacement parameters (Å^2 × 10^3) for fvs9r1.

	**x**	**y**	**z**	**U(eq)**
H(3A)	3660(40)	4370(30)	1799(11)	52(8)
H(4A)	2620(40)	4750(30)	610(12)	66(9)
H(5A)	2260(30)	7770(20)	140(9)	31(6)
H(3B)	4266	6492	2160	20
H(8A)	2420	10571	908	19
H(9A)	3810	12037	1592	21
H(11A)	7785	10582	546	24
H(13A)	9220	7618	1999	52
H(13B)	9742	7072	1419	52
H(13C)	7705	6793	1658	52
H(15A)	8121	14567	394	55
H(15B)	6058	14827	618	55
H(15C)	6345	14242	29	55
H(17A)	-2059	12906	1644	56
H(17B)	-1245	12630	2226	56
H(17C)	-2120	11389	1891	56
